# Knowledge of risk factors and early detection methods toward breast cancer among healthcare workers in Kinshasa, Democratic Republic of the Congo

**DOI:** 10.1002/cnr2.2067

**Published:** 2024-04-10

**Authors:** Stanislas Maseb'a Mwang Sulu, Olivier Mukuku, Arnold Maseb Sul Sulu, Bienvenu Lebwaze Massamba, Stanislas Okitotsho Wembonyama

**Affiliations:** ^1^ Department of Oncology Nganda Hospital Center Kinshasa Democratic Republic of the Congo; ^2^ Department of Research Institut Supérieur des Techniques Médicales de Lubumbashi Lubumbashi Democratic Republic of the Congo; ^3^ Faculty of Medicine University of Kinshasa Kinshasa Democratic Republic of the Congo; ^4^ Faculty of Medicine University of Lubumbashi Lubumbashi Democratic Republic of the Congo

**Keywords:** breast cancer, clinical breast examination, healthcare worker, knowledge, risk factor, screening

## Abstract

**Background:**

Breast cancer (BC) is the most prevalent cancer among women, and it typically presents late in developing countries like the Democratic Republic of the Congo (DRC), leading to higher mortality rates. Late detection at advanced stages of breast cancer can be attributed to the absence of appropriate screening programs and low levels of awareness.

**Aims:**

To evaluate the level of BC knowledge among healthcare workers (HCWs) and identify determinants of good BC knowledge.

**Methods and results:**

An analytical cross‐sectional survey was conducted from March 1 to 31, 2022 involving HCWs practicing in Kinshasa, DRC. Data were collected using a questionnaire administered through direct interviews. Bivariate and multivariate regression techniques were applied. The study interviewed 543 HCWs, with a median age of 35 years (interquartile range: 29–43). Of these, 61% had good BC knowledge, while 39% had poor BC knowledge. Multivariate analysis revealed that HCWs aged 50 years and over (adjusted odds ratio [aOR] = 2.3 [1.2–4.5]), female HCWs (aOR = 1.8 [1.1–2.4]), HCWs working in public healthcare facilities (aOR = 1.5 [1.1–2.5]), and HCWs who had received training on BC (aOR = 1.9; 95% CI: 1.5–3.3) were determinants of good BC knowledge.

**Conclusion:**

This study found that 61% of the surveyed HCWs had good BC knowledge. However, there is still room for improvement in terms of knowledge dissemination. Therefore, it is important to implement continuing medical education programs that focus on raising awareness and improving BC knowledge among HCWs.

## 1 INTRODUCTION

Breast cancer (BC) is the most prevalent cancer in women worldwide,^1^ including the Democratic Republic of the Congo (DRC).^2^ Despite significant advances in the treatment of BC, the outcome is still desperate in low‐income countries, including the DRC.^3^, ^4^ Late diagnosis is a primary reason for the poor prognosis. Early detection of BC is associated with better prognosis, reduced morbidity, and mortality.^5^ Late‐stage presentation and limited access to screening and treatment services contribute to elevated mortality rates associated with BC. Early detection, through increased awareness of risk factors and available screening methods, is paramount in mitigating the burden of this disease. The goal of the World Health Organization's (WHO) new global initiative to combat BC is to reduce mortality from BC worldwide by 2.5% per year, thereby averting 2.5 million deaths from BC worldwide between 2020 and 2040. Three key components are needed to achieve this goal: BC comprehensive management, prompt diagnosis, and promoting health for early detection.^6^ Measures should therefore be taken to achieve this objective. Educating healthcare workers (HCWs), and the community at large about the early warning signs and symptoms of BC and learning about screening methods are crucial components of early diagnosis.^7^ However, it should also be emphasized that effective cancer prevention requires HCWs to take greater account of the various risk factors, through frequent public awareness campaigns.^8^ Previous researches have demonstrated that both HCWs and patients have inadequate knowledge regarding BC associated factors.^7^, ^9^, ^10^ Other studies have reported misconceptions among HCWs (such as the belief that prayer can make BC disappear,^11^ or the belief that keeping money in the bra causes BC^12^). HCWs, being at the forefront of healthcare delivery, play a pivotal role in disseminating crucial information about BC to the community. Their knowledge and awareness not only influence their practices but also contribute significantly to public health initiatives. Good knowledge and awareness are essential for early diagnosis and effective prevention. HCWs have the power to favorably affect patients' attitudes and beliefs regarding screening procedures and to significantly affect their overall perspective on them.^13^ HCWs are often the primary point of contact, not only for their patients but also for parents and relatives, to provide correct advice and information about BC and its screening. Thus, educating HCWs about BC and its screening is an important target for future interventions aimed at improving BC outcomes in the DRC. Understanding the baseline knowledge of HCWs in Kinshasa regarding BC risk factors and early detection methods is essential for designing targeted interventions. Furthermore, to the best of our knowledge, no research has been done in Kinshasa, or anywhere else in the DRC, specifically on the assessment of BC knowledge among HCWs.

This study aims to assess the knowledge of HCWs regarding BC and to identify factors associated with a good level of BC knowledge that could be targeted for future educational interventions. Understanding the current understanding and awareness within this vital demographic can help identify potential gaps and challenges that may hinder effective BC prevention and early diagnosis strategies. The findings of this study have the potential to inform tailored educational programs for HCWs, thereby enhancing their ability to convey accurate and impactful information to the broader community.

## 2 MATERIALS AND METHODS

### 2.1 Study site and population

This analytical cross‐sectional study was conducted in Kinshasa, the capital of the DRC located in the south‐western part of the country (longitude 15°18′48″ East and latitude 4°19′39″ South). The city covers an area of 9965 km^2^ and has an estimated population of 17 million in 2021. Kinshasa is divided into 35 health zones (ZS) and serves as a hub for health services for the entire country and neighboring provinces.

All doctors and nurses from the city's various healthcare facilities were judged eligible for this study, and they were chosen at random. An HCW was excluded from the study if they worked in a healthcare facility that had not consented to participate, or if they were on vacation or absent from work on the day of data collection. Private clinics, general referral hospitals, and health centers were all included in the study. Between March 1 and 31, 2022, data were collected.

A minimum sample size of 317 was determined by random sampling using the formula: *n* = *z* ^2^ *pq*/*d* ^2^, with a 95% confidence interval (95% CI) standard deviation (1.96), a prevalence of good BC knowledge in Congolese women in Kinshasa of 22.09%,^14^ a precision error of 5%, and a non‐response rate of 20%. The study randomly selected 10 health zones (HZs) from a list of 35 HZs in Kinshasa in the first stage. In the second stage, different HCWs were randomly selected and interviewed for the study.

Out of the 600 questionnaires distributed, 557 were returned, and 543 of the returned questionnaires were found to be complete and included in analysis. Thus, the response rate was 90.5%.

### 2.2 Data collection and study variables

The study was conducted from March 1 to 31, 2022, and utilized a semi‐structured questionnaire that was developed from a literature review.^7^ The questionnaire was pre‐tested with 20 HCWs to ensure clarity of meaning and appropriate use of language, taking into account expert opinion. The face‐to‐face interviews were conducted by final‐year medical students who received orientation on the study protocol and questionnaire administration skills. The interviews were supervised by the principal investigator (SMMS).

Ten interviewers were recruited and provided with mock survey tests, a detailed interpretation of the survey questionnaire, and training in the context of the current survey. Before asking participants to complete the questionnaire, the researchers gave them an explanation of the study's goal and methodology and got their signed informed consent. Participants' responses to the survey took an average of 15–20 min. Written informed consent was obtained from all eligible participants for the interview after they were informed about the study's purpose.

After conducting a comprehensive literature review, we developed survey questions and organized the questionnaire into four parts.

The first part comprised socio‐demographic and professional characteristics: age (20–29, 30–39, 40–49, or ≥50 years), gender (female or male), type of healthcare facility (private clinic or public hospital), medical qualification (physician or nurse), number of years of professional practice (<5, 5–9, or ≥10 years), having received training on BC in the 24 months preceding the survey (yes or no), and having ever had a family member or relative who had suffered from BC (yes or no).

The second section comprised the questions on BC knowledge, categorized into four sections: signs and symptoms, potential risk factors, and screening methods.

For the second part, HCWs were asked to respond with either “yes,” “no,” or “I do not know.” Responses were scored dichotomously, with zero point given for incorrect or “I do not know” responses and one point given for each correct answer. The total score was calculated by adding up the points obtained, with a maximum score of 29. Scores were then categorized as good knowledge (18–29 points, 60% or more of correct answers) or either poor knowledge (0–17 points, less than 60% of correct answers).

The third part aimed to evaluate the knowledge of respondents on breast self‐examination (BSE). Participants were asked if they were aware of BSE and whether they believed it was beneficial for the early detection of BC. Other questions related to BSE included at what age should BSE be started, how often should the BSE be carried out, what is the best time to do BSE, who should carry out BSE, and in the event of a breast abnormality during BSE, what action would you recommend.

The final part evaluated the knowledge of clinical breast examination (CBE) and mammography.

The Cronbach's alpha coefficient calculated for the breast cancer (BC) knowledge and screening items yielded an internal consistency reliability of 0.895.

### 2.3 Statistical analysis

Microsoft excel was used to enter and encode the data. STATA version 16.0 was used for the statistical analyses.

To perform a descriptive analysis, we computed medians with interquartile ranges (IQR) for non‐normally distributed quantitative variables, as confirmed by the Shapiro test, and proportions for qualitative variables (frequencies, percentages, and Wilson's 95% CI). To compare the medians across various variable categories, we employed the Mann–Whitney U test or, when advised, the ANOVA test.

In this study, we considered good knowledge as our dependent variable. To find the variables linked to good BC knowledge, we first performed a bivariate analysis using the Chi‐squared test and stepwise method for multiple logistic regression. The regression model contained explanatory variables with a bivariate test value of 0.25. *p‐*values less than .05 were considered as statistically significant.

### 2.4 Ethical considerations

Before the interviews, participants gave their informed consent, and there was no payment or other incentive given to them as part of the study. Participants received guarantees that the information they provided would be kept private and that they could leave the study at any time, for any reason.

## 3 RESULTS

### 3.1 Socio‐demographic characteristics of participants

A total of 543 HCWs completed the survey, of whom 192 (35.4%) were physicians and 351 (64.6%) were nurses. The median age (IQR) of the participants was 35 years (29–43). Over a third (35.4%) of respondents were aged between 30 and 39, and 67.6% were male. A third (32.2%) of respondents worked in private healthcare facilities and 67.8% in public healthcare facilities. Respondents had been in clinical practice experience for a median of 5 years (3–10) and only 32.4% had reported receiving a training session on BC in the 24 months preceding the survey. Two hundred and eighteen (40.2%) participants reported having a family member or close friend who had ever suffered from BC (Table 1).

**TABLE 1:** Demographic characteristics of respondents with median knowledge scores.

Variable	*N* = 543 [*n* (%)]	Median (interquartile range) of knowledge score	*p*‐value
Age			
20–29 years	148 (27.3)	18.5 (14.0–22.0)	.0014
30–39 years	192 (35.4)	20.0 (15.0–24.0)	
40–49 years	124 (22.8)	18.5 (14.0–23.0)	
≥50 years	79 (14.5)	22.0 (18.0–25.0)	
Gender			
Female	176 (32.4)	21.0 (16.0–24.0)	.0002
Male	367 (67.6)	19.0 (14.0–22.0)	
Medical qualification			
Physician	192 (35.4)	20.5 (16.0–24.0)	.0004
Nurse	351 (64.6)	19.0 (14.0–23.0)	
Type of health facility			
Private clinic	175 (32.2)	19.0 (14.0–23.0)	.1440
Public hospital	368 (67.8)	20.0 (15.0–23.0)	
Years of clinical practice			
<5 years	229 (42.2)	19.0 (14.0–23.0)	.4378
5–9 years	156 (28.7)	19.0 (15.0–23.0)	
≥10 years	158 (29.1)	20.0 (15.0–23.0)	
Have received training on breast cancer in the last 24 months			
Yes	176 (32.4)	20.0 (17.0–24.0)	.0001
No	367 (67.6)	19.0 (14.0–23.0)	
Have a family member or relative who has suffered from breast cancer			
Yes	218 (40.2)	19.0 (15.0–23.0)	.5420
No	307 (59.8)	20.0 (14.0–23.0)	

### 3.2 Participants' knowledge of breast cancer

The median knowledge score was 19 (15–23) out of a total of 29 points. Table 1 shows that respondents aged 50 or over, female respondents, physicians, and those who had received training on BC had significantly higher median scores than others (*p* < .05). We classified a score of 0–17 as a poor of BC knowledge and a score ≥18 as a good BC knowledge. Of the participants, 331 (61.0%) had a good level and 212 (39.0%) had a poor level of BC knowledge (Table 1).

More than 70% of the respondents identified a family history of BC, oral contraceptive use, and radiation exposure as potential risk factors for developing BC. However, only around 50% recognized smoking, alcohol consumption, a diet rich in animal fats, late first pregnancy, and obesity as potential risk factors for BC.

In the section on signs and symptoms of BC, the majority of participants recognized that a change in breast shape (asymmetry) and breast size could be signs of BC, with 85.5% and 74.4% of participants respectively indicating this. Over 81% and 77.4% of respondents respectively recognized abnormal breast discharge and a painful or painless breast lump as signs of BC. Breast ulceration and pain were recognized as signs of BC by 83% of respondents.

Regarding the methods used to diagnose BC, more than 70% of participants gave the correct answer for anatomopathological examination of breast tissue (70.5%) and BSE (75.9%). Mammography was recognized as a method of detecting BC by 80.5% of respondents, while only 63.9% recognized CBE and 36.8% recognized ultrasound (Table 2).

**TABLE 2:** Percentage of correct answers to questions relating to participants' knowledge of breast cancer.

Items assessing knowledge about breast cancer	Number (*n* = 543)	Percentage	95% confidence interval
Potential risk factors for developing breast cancer			
Race/ethnicity	213	39.2	35.2–43.4
Advanced age (>40 years)	326	60.0	55.9–64.1
Family history of breast cancer	413	76.1	72.3–79.5
Larger breasts	411	75.7	71.9–79.1
High‐fat diet	275	50.6	46.5–54.8
Smoking	304	56.0	51.8–60.1
Oral contraception	385	70.9	67.0–74.6
Radiation exposure	395	72.7	68.9–76.3
Alcohol consumption	273	50.3	46.1–54.5
Late first pregnancy (>30 years)	262	48.3	44.1–52.5
Early onset of menarche (<12 years)	174	32.0	28.3–36.1
Late menopause (>55 years)	202	37.2	33.2–41.3
Lack of physical activity	191	35.2	31.3–39.3
Obesity (post‐menopause)	255	47.0	42.8–51.2
Signs and symptoms related to breast cancer			
Lump in the breast	420	77.4	73.6–80.7
Abnormal discharge from the nipple	442	81.4	77.9–84.5
Change in the size of the breast	404	74.4	70.6–77.9
Change in the shape of the breast	464	85.5	82.2–88.2
Inversion/pulling in of the nipple	362	66.7	62.6–70.5
Lump under armpit	431	79.4	75.8–82.6
Ulceration of the breast	455	83.8	80.5–86.7
Pain or sorensse in the breast	451	83.1	79.7–86.0
Discoloration of the breast	422	77.7	74.0–81.0
Weight loss	340	62.6	58.5–66.6
Diagnosis methods for breast cancer			
Pathological examination of breast tissue	383	70.5	66.6–74.2
Self‐breast examination	412	75.9	72.1–79.3
Clinical breast examination by physician	347	63.9	59.8–67.8
Mammography	437	80.5	76.9–83.6
Ultrasound	200	36.8	32.9–41.0
Level of knowledge based on the total score			
Poor (score 0–17)	212	39.0	35.0–43.2
Good (score 18–29)	331	61.0	56.8–65.0
Median (interquartile ranges) for the knowledge scale	19.0	(15.0–23.0)	

### 3.3 Breast self‐examination's knowledge

Table 3 shows the results regarding knowledge and practice of BSE. In general, 484 (89.1%) participants recognized BSE as an effective tool for the early detection of BC. Among them, 267 (49.2%) had received BSE training. A total of 200 (36.8%) participants chose to start BSE at puberty, while 122 (22.5%) preferred to start at the age of 20. The majority (50.1%) agreed that 1 week after menstruation is the best time to practice BSE, and 282 participants (51.9%) thought BSE should be done daily. Less than half (49.2%) knew that BSE should be done by the person herself, and 438 (80.7%) said that in the event of an abnormality, a physician should be consulted. Finally, the vast majority (88.2%) agreed that BSE is a good practice.

**TABLE 3:** Knowledge and practice of breast self‐examination (BSE).

Questions/statements to assess knowledge of BSE	Number (*n* = 543)	Percentage	95% confidence interval
Is BSE a useful tool for early detection of BC?			
Yes	484	89.1	86.2–91.5
No	59	10.9	8.5–13.8
Have you ever had any training on BSE?			
Yes	267	49.2	45.0–53.4
No	276	50.8	46.6–55.0
At what age should BSE be started?			
From birth	4	0.7	0.3–1.9
From puberty	200	36.8	32.9–41.0
From 20 years	122	22.5	19.2–26.2
From 30 years	100	18.4	15.4–21.9
After menopause	11	2.0	1.1–3.6
I do not know	106	19.5	16.4–23.1
How often should the BSE be carried out?			
Daily	282	51.9	47.7–56.1
Weekly	94	17.3	14.4–20.7
Monthly	78	14.4	11.7–17.6
Yearly	11	2.0	1.1–3.6
I do not know	78	14.4	11.7–17.6
What is the best time to do BSE?			
During menstrual flow	66	12.2	9.7–15.6
A week after menstraul period	272	50.1	45.9–50.3
During pregnancy	7	1.3	0.6–2.6
During breastfeeding	6	1.1	0.5–2.4
I do not know	192	35.4	31.5–39.5
Who should carry out BSE?			
Physician	38	7.0	5.1–9.5
Training nurse	4	0.7	0.3–1.9
The individual	267	49.2	45.0–53.4
All of the above	145	26.7	23.2–30.6
I do not know	89	16.4	13.5–19.7
How is BSE performed?			
By inspecting the breast in the mirror	18	3.3	2.1–5.2
By feeling the breast with the hand	228	42.0	37.9–46.2
By feeling the armpit with the hand	24	4.4	3.0–6.5
By doing ultrasound of the breast	13	2.4	1.4–4.1
By mammography	65	12.0	9.5–15.0
All of the above	66	12.2	9.7–15.2
I do not know	129	23.8	20.4–27.5
In the event of a breast abnormality during BSE, what action would you recommend?			
Leave it to god and pray	12	2.2	1.3–3.8
Do some laboratory tests	5	0.9	0.4–2.1
See a physician	438	80.7	77.1–83.8
All of the above	37	6.8	5.0–9.3
I do not know	51	9.4	7.2–12.1
In your opinion, is BSE a good practice?			
Yes	479	88.2	85.2–90.7
No	64	11.8	9.3–14.8

### 3.4 Clinical breast examination's knowledge

The findings concerning knowledge and practice of CBE are illustrated in Table 4. 88.4% (480) of participants recognized the effectiveness of CBE in detecting BC. Among the respondents, 38.0% (208) preferred to have an HCW conduct the CBE, 26.1% (142) believed that mammography should be included in CBE, 25.1% (136) recommended performing CBE monthly, and 14.5% (79) yearly intervals.

**TABLE 4:** Knowledge and practice of clinical breast examination (CBE).

Questions/statements to assess knowledge of CBE	Number (*n* = 543)	Percentage	95% confidence interval
Is CBE a useful tool for early detection of BC?			
Yes	480	88.4	85.4–90.8
No	63	11.6	9.2–14.6
Who should carry out CBE?			
Physician	208	38.3	34.3–42.5
Training nurse	10	1.8	1.0–3.4
The individual	16	3.0	1.8–4.7
All of the above	131	24.1	20.7–27.9
I do not know	178	32.8	29.0–36.8
CBE is done using			
Ultrasound of the breast	26	4.8	3.3–6.9
Hand	227	41.8	37.7–46.0
Mammography	142	26.1	22.6–30.0
All of the above	85	15.7	12.8–19.0
I do not know	63	11.6	9.2–14.6
How often should CBE be carried out?			
Daily	160	29.5	25.8–33.4
Weekly	54	9.9	7.7–12.8
Monthly	136	25.1	21.6–28.9
Yearly	79	14.5	11.8–17.8
I do not know	114	21.0	17.8–24.6

### 3.5 Mammography's knowledge

A majority of the participants, 469 (86.4%), acknowledged mammography as an effective tool for the early detection of BC. Out of these, 354 (65.2%) correctly identified mammography as a radiological examination. 231 (42.5%) agreed that mammography should be initiated at the age of 40, while 210 (38.7%) mentioned that mammography should be performed when a lump is detected during BSE or CBE (as presented in Table 5).

**TABLE 5:** Knowledge and use of mammography.

Questions/statements to assess knowledge of mammography	Number (*n* = 543)	Percentage	95% confidence interval
Is mammography a useful tool for early detection of BC?			
Yes	469	86.4	83.2–89.0
No	74	13.6	11.0–16.8
Have you ever had training in mammography?			
Yes	119	21.9	18.6–25.6
No	424	78.1	74.4–81.4
What is mammography?			
An ultrasound examination	91	16.8	13.9–20.1
A manual examination	11	2.0	1.1–3.6
An X‐ray examination	354	65.2	61.1–69.1
All of the above	32	5.9	4.2–8.2
I do not know	55	10.1	7.9–13.0
At what age should mammography be started?			
From birth	2	0.4	0.1–1.3
From puberty	105	19.3	16.2–22.9
From 20 years	67	12.3	9.8–15.4
From 40 years	231	42.5	38.5–46.7
After menopause	23	4.2	2.8–6.3
All of the above	10	1.8	1.0–3.4
I do not know	105	19.3	16.2–22.9
How often should mammography be carried out?			
Weekly	78	14.4	11.7–17.6
Monthly	76	14.0	11.3–17.2
Yearly	75	13.8	11.2–17.0
Only when a lump is found during breast self‐examination or clinical breast examination	210	38.7	34.7–42.8
I do not know	104	19.2	16.1–22.7

### 3.6 Determinants of participants' good knowledge of breast cancer

Table 6 presents the results of the bivariate analyses conducted to determine the association between the participants' sociodemographic and professional characteristics and their level of BC knowledge. The study found significant associations between good BC knowledge and variables such as age, gender, medical title, and receiving training on BC within the 24 months before the survey.

**TABLE 6:** Bivariate analysis of the breast cancer knowledge level.

Variable	BC knowledge level		Unadjusted odds ratio [95% confidence interval]	*p*‐value
	Good (*n* = 331) *n* (%)	Poor (*n* = 212) *n* (%)		
Age				
20–29 years	83 (56.1)	65 (43.9)	1.0	
30–39 years	117 (60.9)	75 (39.1)	1.2 [0.8–1.9]	.429
40–49 years	69 (55.6)	55 (44.4)	1.0 [0.6–1.6]	1.000
≥50 years	62 (78.5)	17 (21.5)	2.9 [1.5–5.3]	.001
Gender				
Female	121 (68.8)	55 (31.2)	1.6 [1.1–2.4]	.013
Male	210 (57.2)	157 (42.8)	1.0	
Medical qualification				
Physician	133 (69.3)	59 (30.7)	1.7 [1.2–2.5]	.004
Nurse	198 (56.4)	153 (43.6)	1.0	
Type of health facility				
Private clinic	97 (55.4)	78 (44.6)	1.0	
Public hospital	234 (63.6)	134 (36.4)	1.4 [0.9–2.0]	.084
Years of clinical practice				
<5 years	137 (59.8)	92 (40.2)	1.0	
5–9 years	93 (59.6)	63 (40.4)	1.0 [0.6–1.5]	1.000
≥10 years	101 (63.9)	57 (36.1)	1.2 [0.8–1.8]	.479
Have received training on breast cancer in the last 24 months				
Yes	128 (72.7)	48 (27.3)	2.2 [1.5–3.2]	.0001
No	203 (55.3)	164 (44.7)	1.0	
Have a family member or relative who has suffered from breast cancer				
Yes	131 (60.1)	87 (39.9)	0.9 [0.7–1.3]	.803
No	200 (61.5)	125 (38.5)	1.0	

**FIGURE 1:**
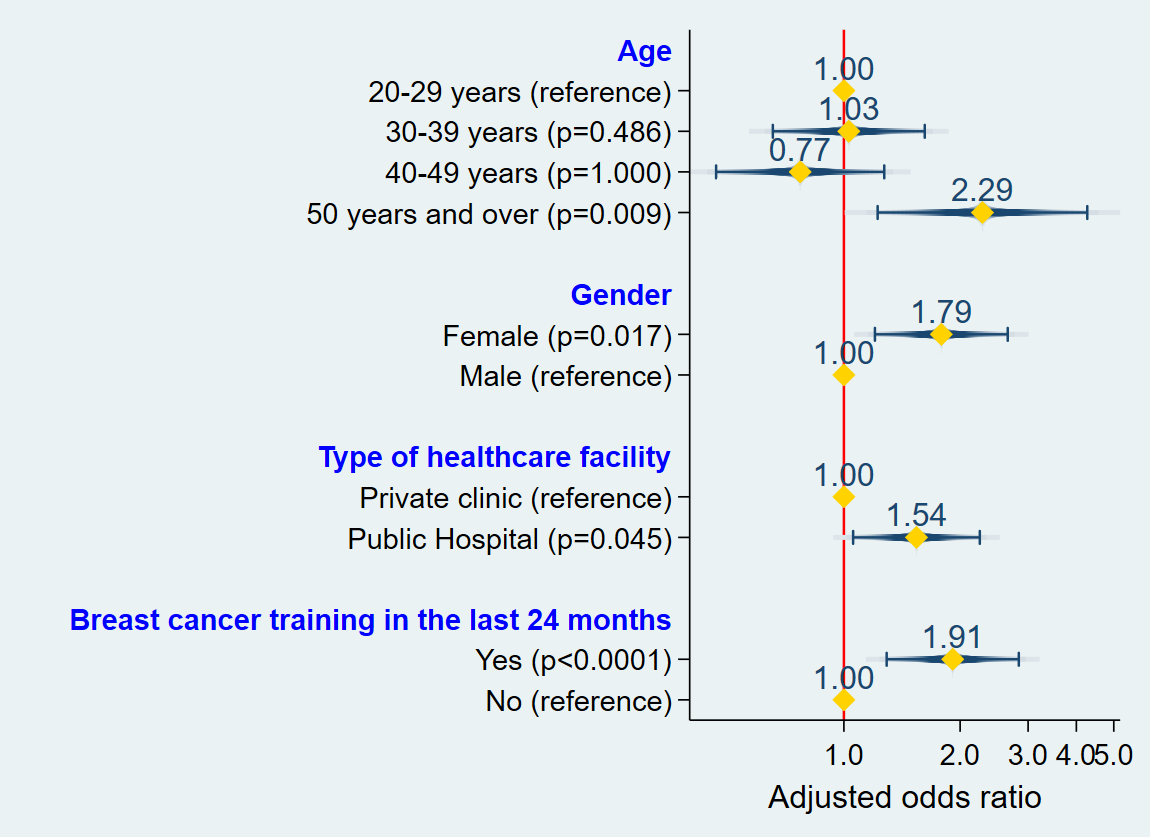
Logistic regression of good breast cancer knowledge's determinants.

The logistic regression revealed that respondents aged ≥50 years had higher chances of having good BC knowledge compared to others (adjusted odds ratio [aOR] = 2.3; 95% CI: 1.2–4.5; *p* = .009). Furthermore, female participants were significantly more likely to have good knowledge than their male counterparts (aOR = 1.8; 95% CI: 1.1–2.4; *p* = .017). Respondents working in public healthcare facilities had higher chances of having good BC knowledge compared to those working in private clinics (aOR = 1.5; 95% CI: 1.1–2.5; *p* = .045). The results also showed that respondents who received training on BC had significantly higher odds of having good BC knowledge than those who did not receive training (aOR = 1.9; 95% CI: 1.5–3.3; *p* < .001) (Figure 1).

## 4 DISCUSSION

For BC to be detected early and treated as effectively as possible, awareness and knowledge are essential. The level of BC knowledge among HCWs is a significant factor in the optimal management of their patients. It is obvious that when HCWs actively participate in health education and act as positive role models for the community, health will improve.^15^ This study, the first of its kind, aimed to evaluate the knowledge of BC and its screening among HCWs practicing in Kinshasa.

The study found that 61% (95% CI: 56.8%–65.0%) of the participants had good BC knowledge, particularly concerning its risk factors, signs and symptoms, and diagnostic methods. Compared to other studies,^7^, ^12^, ^15^, ^16^, ^17^ the rate of good BC knowledge among our cohort appears to be higher. However, despite this, this research emphasizes the necessity of further education and training on BC for HCWs, given that only 32.4% of respondents had received training on BC in the previous 24 months. Such training should aim to improve the knowledge of HCWs in our setting, which will ultimately lead to better health outcomes for patients.

The study revealed that HCWs in our cohort were most familiar with a family history of BC, radiation exposure, and oral contraceptive use as risk factors for BC, while early menarche, late menopause, and physical inactivity were the least well‐known. These results are in line with previous researches done on HCWs in various countries.^7^, ^15^, ^18^ Based on our findings, we recommend that training HCWs should emphasize the risk factors for BC, particularly those that are less well known. Despite being the key to BC awareness and screening, most studies have shown that HCWs have poor knowledge and practice of these early detection methods.^17^, ^19^

BC risk factors' knowledge among HCWs is crucial to providing adequate screening advice to women with a high‐risk profile, particularly in the DRC where BC screening is not yet nationalized. Acquiring knowledge about BC is crucial to preventing and controlling this disease, and making informed decisions about interventions. An ill‐informed healthcare workforce may impede raising awareness in the general population.^7^ Thus, it is important to strengthen the education of HCWs to acquire basic knowledge about BC, to help those affected get diagnosed and managed early, and to improve their quality of life.

Our findings regarding BSE were encouraging, as the majority of participants (89.1%) acknowledged its significance. They also had good BSE knowledge. While there has been discussion recently regarding the suitability of BSE as an early detection instrument for BC, BSE plays a crucial role in the DRC in areas where mammography may not be accessible due to financial constraints. Moreover, statistics show that 90% of breast lumps are found by women themselves.^7^

In this study, a very small proportion of providers, only 2.2%, stated that they would advise their patients to leave it to God and pray in the event of a breast abnormality during BSE. Cultural and religious misconceptions about BC could be significant factors contributing to the late presentation of the disease.^20^ For instance, in a Nigerian study, 17.6% of female HCWs surveyed believed in the efficacy of prayer and 43.4% believed in the efficacy of traditional or herbal therapy, which the authors believe could have a negative effect on appropriate BC awareness, thus contributing to delayed diagnosis.^11^

After conducting multiple logistic regression, this study identified significant associations between a good level of BC knowledge and the following variables: age ≥50 years (aOR = 2.3; 95% CI: 1.2–4.5; *p* = .009), female gender (aOR = 1.8; 95% CI: 1.1–2.4; *p* = .017), receiving training on BC in the 24 months before the survey (aOR = 1.9; 95% CI: 1.5–3.3; *p* < .001), and working in a public healthcare facility (aOR = 1.5; 95% CI: 1.1–2.5; *p* = .045). The proportion of respondents with good BC knowledge was significantly higher in the age group of ≥50 years compared to other age groups. A similar observation was made by Nguefack et al.^21^ in Douala (Cameroon). We believe that respondents aged 50 and over had acquired experience by consulting a large number of patients with BC during their clinical practice, although the number of years of clinical practice was not associated with a good level of BC knowledge.

Female HCWs were more likely to have good BC knowledge than male HCWs. This may be due to the fact that women are more likely to perceive that they are at risk of developing the disease during their lifetime than men, which would lead to an improvement in their BC knowledge and its detection.

HCWs working in public healthcare facilities were approximately twice as likely to have good BC knowledge than those in private facilities. This finding highlights the need for further research to understand how training is organized within these two sectors. It is worth noting that a Pakistani study did not find any significant difference in knowledge between HCWs in the public and private sectors.^22^

The present study shows the importance of conducting more structured training campaigns with practical sessions, if necessary, among HCWs, as we found that having received BC training was a predictor of a good level of knowledge about BC. According to the WHO, public health education measures aimed at making women and their families aware of the BC signs/symptoms, and the importance of early detection and appropriate treatment, contribute to more women consulting an HCW as soon as BC is suspected and before any cancer reaches an advanced stage.^6^ These advances are possible even in the absence of mammography, a technique that is currently difficult to implement in a large number of countries. Public education must go hand in hand with education for HCWs about the signs/symptoms of early‐stage BC, so that women can be referred for diagnosis if necessary.^6^

The study examines BC knowledge among HCWs in Kinshasa, highlighting its limitations, strengths, and hypotheses. Among the limitations, the cross‐sectional design hinders causality and generalizability, while random sampling and self‐reporting of data pose potential issues of selection and memory bias. However, the large sample size enhances reliability, and the study establishes a strong foundation for future research. Strengths include a comprehensive assessment of breast cancer knowledge among healthcare professionals, providing valuable data for targeted interventions. The implicit hypothesis is that improving breast cancer knowledge among healthcare professionals can lead to more effective screening and management practices.

## 5 CONCLUSION

This study revealed that 61% of HCWs had good knowledge of BC, with age ≥50 years, female gender, public healthcare facility, and receiving training on BC in the last 24 months before the survey being significant determinants of good BC knowledge. Based on these findings, we suggest continuous and efficient BC training for HCWs. Further studies on HCWs from other regions of the DRC can provide insights to enhance the comprehension of BC awareness among Congolese HCWs. The future prospects of this study include exploring the long‐term impacts of training programs, identifying determinants of awareness among HCWs, and collaborating with other stakeholders to enhance public health.

## AUTHOR CONTRIBUTIONS

**Olivier Mukuku:** Conceptualization; methodology; software; formal analysis; writing – original draft. **Stanislas Maseb'a Mwang Sulu:** Conceptualization; investigation; resources; validation; project administration; writing – review and editing; data curation. **Arnold Maseb Sul Sulu:** Resources; project administration; data curation; investigation; visualization. **Bienvenu Lebwaze Massamba:** Supervision; investigation; writing – review and editing; validation. **Stanislas Okitotsho Wembonyama:** Writing – review and editing; supervision; conceptualization; methodology; visualization.

## CONFLICT OF INTEREST STATEMENT

The authors have stated explicitly that there are no conflicts of interest in connection with this article.

## Data Availability

The data that support the findings of this study are available from the corresponding author upon reasonable request.
